# Hyperoxia Inhibits Proliferation of Retinal Endothelial Cells in a Myc-Dependent Manner

**DOI:** 10.3390/life11070614

**Published:** 2021-06-25

**Authors:** Charandeep Singh, Andrew Benos, Allison Grenell, Sujata Rao, Bela Anand-Apte, Jonathan E. Sears

**Affiliations:** 1 Ophthalmic Research, Cole Eye Institute, Cleveland Clinic, Cleveland, OH 44195, USA; CSINGH3@mgh.harvard.edu (C.S.); benosa@ccf.org (A.B.); abg77@case.edu (A.G.); raos7@ccf.org (S.R.); anandab@ccf.org (B.A.-A.); 2 Department of Pharmacology, Case Western Reserve University School of Medicine, Cleveland, OH 44106, USA; 3 Department of Ophthalmology and Department of Molecular Medicine, Cleveland Clinic Lerner College of Medicine at Case Western Reserve University, Cleveland, OH 44195, USA; 4 Cardiovascular and Metabolic Sciences, Cleveland Clinic, Cleveland 44195, OH, USA

**Keywords:** Myc, hyperoxia, ROP, retinoblastoma, cell cycle

## Abstract

Oxygen supplementation is necessary to prevent mortality in severely premature infants. However, the supraphysiological concentration of oxygen utilized in these infants simultaneously creates retinovascular growth attenuation and vasoobliteration that induces the retinopathy of prematurity. Here, we report that hyperoxia regulates the cell cycle and retinal endothelial cell proliferation in a previously unknown Myc-dependent manner, which contributes to oxygen-induced retinopathy.

## 1. Introduction

Retinopathy of prematurity (ROP) is a leading cause of infant blindness world-wide, accounting for 184,700 new cases annually [1,2,3]. Although oxygen supplementation is necessary to prevent mortality in premature infants, an oxygen-rich environment in severely low birthweight infants can be detrimental to the developing premature organs, such as the retina, brain, and lung. Although ROP does not develop until the corrected gestational age of 30–32 weeks, retinovascular growth attenuation and vasoobliteration caused by higher than in utero oxygen concentrations creates increased avascular retinal tissue that causes pathological angiogenesis, followed by retinal detachment and blindness [4]. One of the early clinical signs of ROP is retinovascular growth suppression [5,6,7]. A similar phenotype can be recapitulated in the mouse and rat model of oxygen-induced retinopathy (OIR) [8,9]. This phenotype is often referred to as “oxygen toxicity”, as it bears the negative connotation reflecting the ill effects of oxygen on the vascular development. In vitro and in vivo studies have demonstrated that hyperoxia increases the formation of reactive oxygen and nitrogen species [10,11]. Furthermore, hyperoxia upregulates neuronal apoptosis in the brain and the retina [12,13,14,15,16]. Although neurons are nonmitotic, fully differentiated cells, they do harbor cell-cycle proteins, and recent studies have demonstrated that dysregulation in cell-cycle protein levels in neurons can lead to apoptosis. However, mitotic cells of nonneuronal origin can enter into a long G_0_ phase under unsuitable circumstances and can re-enter the cell cycle when conditions become favorable [17,18]. In mice, hyperoxia affects the vasculature in early postnatal stages, when endothelial cells are still proliferating and migrating. Smith et al. (1993) demonstrated that once the vasculature is fully developed, these mice do not develop the vasoobliteration and neovascularization phenotype after exposure to 5 days of hyperoxia [8]. This implies that susceptibility to hyperoxia is not merely caused by oxidative damage but involves more complex molecular pathways that are active in the early stages of retinal development. For example, as in oxygen-induced retinopathy, a postnatal oxygen-rich environment inhibits proliferation of cardiomyocytes [19]. Mammalian cardiomyocytes have regenerative capacity at birth but lose this potential postnatally as the oxygen-rich environment prevents cell proliferation. In mice, after postnatal day 7, cardiomyocytes become binucleated and permanently exit the cell cycle through DNA damage-induced cell-cycle arrest [19]. D’Amore and Sweet found downregulation of bovine adrenal cortex endothelial cells in response to hyperoxia in vitro. However, they reported no downregulation of proliferation of cultured retinal pericytes in vitro [20], in contrast to Uno et al. who demonstrated downregulation of proliferation of hyperoxic canine endothelial cells [21]. These differences in response to hyperoxia amongst cell types demonstrate the heterogeneity of cell-cycle control and warrant closer examination of cell-type-specific mechanisms.

Myc is a critical regulator of the cell cycle and cellular proliferation [22]. Hypoxia-induced increase in HIF1 levels result in decreased Myc RNA and protein expression [23,24,25]. Furthermore, Myc levels are inversely proportional to nutrient availability and cell density. Myc is downregulated during starvation conditions, halting the cell cycle, which leads to the loss of proliferation to protect the essential supplies for survival [22]. One of the mechanisms by which Myc upregulates cellular proliferation is via upregulating polyamine production [26]. The polyamine pathway is required for normal proliferation and growth [27,28]. Hypoxia increases glycolysis by upregulating pyruvate dehydrogenase kinase-1 (PDK1), which phosphorylates pyruvate dehydrogenase (PDH) and thereby inhibits entry of glycolytic carbon into the TCA cycle. This switch in metabolic flux downregulates cell proliferation by inducing the expression of cyclin-dependent kinase inhibitor (CDKI). Although phosphorylation of PDH is HIF-dependent, upregulation or downregulation of Myc by HIF or vice-versa is context-dependent, and there have been no studies on effects of hyperoxia on Myc protein levels. HIF can suppress cell proliferation by inhibiting the transcriptional activity of Myc (by destabilizing Myc’s interactions with other transcriptional co-factors). Recent reports have shown that HIF1 displaces Myc from MYC-associated protein X (MAX), resulting in destabilization of Myc [29,30]. These findings appear to contradict the phenotype of OIR; if hyperoxia downregulates HIF, one might assume that Myc would be induced by hyperoxia. In this investigation, we analyzed the effect of hyperoxia on key cell-cycle regulators. Our findings indicate a central effect of hyperoxia on Myc protein levels, providing a molecular mechanism of how an oxygen-rich environment induces cell-cycle arrest in retinal endothelial cells.

## 2. Results

**Figure 1:**
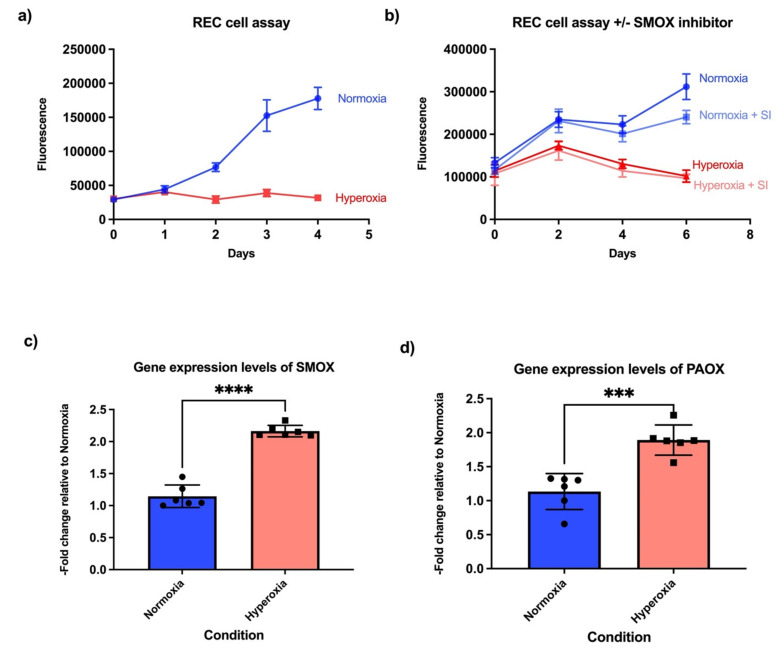
Hyperoxia inhibits proliferation and increases expression of polyamine oxidation genes in retinal endothelial cells. (**a**) Retinal endothelial cells cultured in hyperoxia demonstrated proliferation defects even in the presence of a proprietary medium containing growth factors (Cell Biologics, catalogue number H1168). Blue is normoxic conditions, red hyperoxic conditions (*n* = 8 biological replicates per condition). (**b**) Proliferation defects were not rescued by spermidine oxidase inhibitor (SI) MDL72527 (*n* = 6 biological replicates per condition). (**c**) Gene expression levels of spermine oxidase (SMOX) in normoxia versus hyperoxia. (**d**) Gene expression levels of peroxisomal N (1)-acetyl-spermine/spermidine oxidase (PAOX) in normoxia versus hyperoxia. (*n* = 6 biological replicates per condition). Student’s *t*-test *p*-value *** < 0.001, **** < 0.0001 in all the bar graphs.

To study the effect of hyperoxia on retinal endothelial cell proliferation, we cultured primary human retinal endothelial cells for 24 h under normoxic conditions, followed by hyperoxic or normoxic conditions for 4–6 days. Cellular proliferation was significantly reduced under hyperoxic conditions (Figure 1a), despite the presence of mitogens such as VEGF, IGF, and EGF (please refer to the materials and methods section for media description).

We next examined the expression of polyamine oxidation/breakdown genes, as polyamine levels are critical regulators of cell proliferation in prokaryotes and eukaryotes [31]. Polyamines modulate translation by making complexes with RNA. Critical enzymes in the polyamine pathway, such as ornithine decarboxylase (ODC), peak at G1/S and G2/M transition points, implying that polyamine levels control these checkpoints [32]. In addition, Nakayama and Nakayama (1998) demonstrated that the cell-cycle inhibitors p27Kip1 and p21Cip1/WAF1 are upregulated in response to low polyamine concentration in the cells [33]. This finding was confirmed by Yamashita et al. (2013), who demonstrated that p27Kip1 translation was enhanced by polyamine deficiency [32]. In the retina, which is comprised of multiple cell types, oxidation/breakdown of spermine and spermidine into N-acetylated forms increases with no change in putrescine levels in response to hyperoxia and induces neuronal death [5]. Spermine oxidase (SMOX), an enzyme that catabolizes early substrates of the growth-inducing polyamine pathway, is reported to be increased in hyperoxic conditions [5]. We investigated whether the expression levels of polyamine oxidation genes are regulated at transcriptional levels in response to hyperoxia. Hyperoxia indeed results in upregulation of SMOX in the endothelial cells as compared to normoxia (Figure 1c). We also measured expression of another gene responsible for polyamine oxidation, peroxisomal N (1)-acetyl-spermine/spermidine oxidase (PAOX), and found increased expression in response to hyperoxia (Figure 1d). This implies that the polyamine oxidation genes are transcriptionally controlled in hyperoxic conditions. The SMOX inhibitor MDL 72527 has been shown to reduce retinal neuronal death in the OIR model [5]. We determined that SMOX inhibition could not rescue the cell proliferation phenotype in endothelial cells cultured under hyperoxic conditions (Figure 1b).

**Figure 2:**
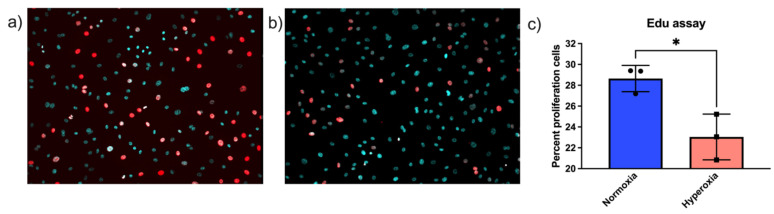
Hyperoxia decreases S-phase Edu-positive endothelial cells. Cells were exposed to (**a**) normoxic or (**b**) hyperoxic conditions for 24 h, following which they were stained with Edu Click-iT^TM^ reagent and simultaneously stained with Hoechst stain. Edu-positive cells are represented in red and Hoechst nuclear staining in cyan. Edu- and Hoechst-stained images were quantified separately using ImageJ, and percentages of proliferating cells were calculated for both the conditions. (**c**) There were higher numbers of proliferating cells in normoxic conditions as compared to hyperoxic conditions (*t*-test *p*-value * < 0.05).

In addition to cell number, we also performed Edu labeling, which labels S-phase cells by incorporating Edu into newly synthesized DNA. Cells were exposed to 24 h of normoxia or hyperoxia, labeled with Edu and analyzed. To count the total number of cells in frame, cells were additionally labeled with Hoechst stain to locate the nuclei of the living cells. There were statistically significantly fewer proliferating cells in hyperoxic conditions compared to normoxic conditions. Edu- and Hoechst-stained merged images of normoxic cells are depicted in Figure 2a and hyperoxic in Figure 2b. Edu- and Hoechst-stained images were merged in Fiji ImageJ in silico [34]. Percentages of Edu-positive cells were quantified using ImageJ and depicted in Figure 2c [35].

**Figure 3:**
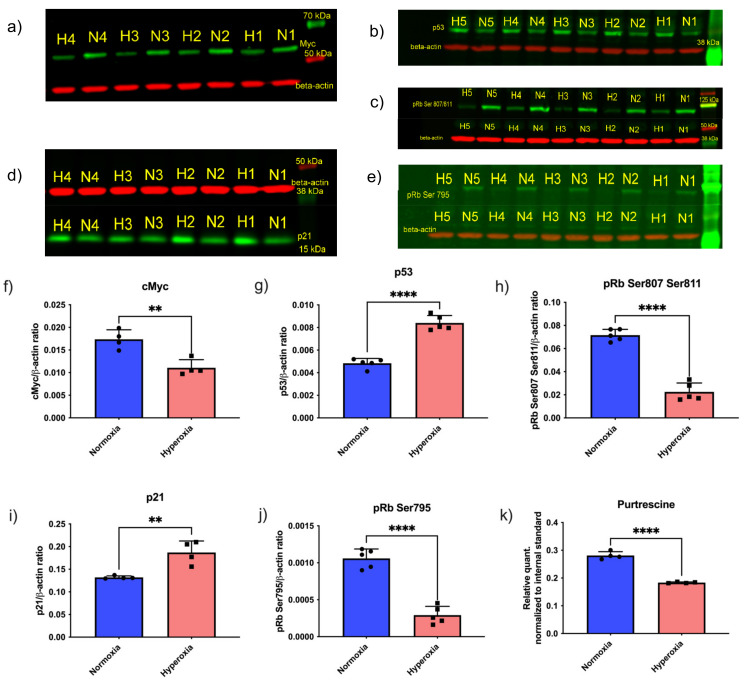
Cell-cycle-signaling proteins, including Myc and downstream proteins, affected by hyperoxia in endothelial cells. Western blots of normoxic (N1–5; each number represent biological replicate) and hyperoxic (H1–5; each number represent biological replicate) samples demonstrate a decrease in cell-cycle proteins that induce growth, and an increase in cell-cycle proteins that inhibit growth by hyperoxia. (**a**) Myc (**b**) p53 (**c**) pRb Ser 807/811 (**d**) p21 (**e**) pRb Ser 795. Quantification of the western blot is provided in the histograms (**f**) Myc, (**g**) p53, (**h**) pRb Ser 807/811, (**i**) p21, and (**j**) pRb Ser 795. (**k**) GC-MS readout of Putrescine in the cells. Each dot in the bar graph represents a biological replicate. Student’s *t*-test *p*-value ** < 0.01, **** < 0.0001 in all the bar graphs. The full pictures of the western blots can be seen in Supplementary Figure S1–S5.

Given that the inhibition of enzymes that downregulate critical polyamines necessary for growth did not rescue the growth of hyperoxic endothelial cells, and putrescine levels were decreased in response to hyperoxia (Figure 3k), we further examined upstream cell-cycle regulators in synchronized primary human retinal endothelial cells. Since Myc protein levels positively correlate with cell proliferation in many different cell types, we measured Myc protein levels in normoxic vs. hyperoxic endothelial cells. Myc protein levels were significantly reduced in hyperoxic endothelial cells compared to normoxic conditions (Figure 3a,f). However, we did not observe any changes in the Myc gene expression between normoxia and hyperoxia (data not shown). To further confirm the relationship between hyperoxia and cell-cycle arrest, we next evaluated p53, because this established regulator of the cell cycle is reported to regulate the phosphorylation of Rb (pRb) [36]. The p53 protein can either activate cell-cycle arrest by inducing p21/Rb axis or apoptosis by inducing the BCL-2 pathway. Despite this duality of function, it is reported that only one of these pathways is activated at a time; however, it is not clear which cellular events determine which of these pathways could be activated [36,37]. We measured p53 and p21 levels in normoxic and hyperoxic conditions. p53 (Figure 3b,g) and p21 (Figure 3d,i) levels were increased in hyperoxic conditions, confirming cell-cycle arrest. Taken together, these findings establish that hyperoxia causes cell-cycle arrest in the G1 phase, via p53 and Myc-dependent pathways.

To further confirm that hyperoxia induces cell-cycle arrest, we evaluated the phosphorylation of retinoblastoma protein (Rb). The first step in committing cells to cell division is transition from the G1 to the S phase, which is dependent on phosphorylation of the retinoblastoma (Rb) protein [22,38]. Phosphorylated Rb leads to the increased concentration of E2F elongation factor, thereby signaling translation of the proteins required for the S-phase [22,38]. There are 19 known phosphorylation sites on human Rb1 protein (source: Uniport) [39]; of these, the three most important sites involved in cell-cycle regulation are Ser 807, Ser 811, and Ser 795 [40]. Recent work by multiple teams have highlighted that out of these three sites, Ser 807 and Ser 811 regulate c-Abl binding of Rb1 [38,40]. Ser795 is involved in binding of Rb1 to E2F transcription factor [38,40]. We measured the levels of phosphorylated pRb Ser807/811 (Figure 3c,h) and pRb Ser795 (Figure 3e,j). Both the phosphorylated forms of Rb protein were decreased in response to hyperoxia, indicating cell-cycle arrest in G1 phase. We additionally measured the levels of putrescine and found it to be decreased in hyperoxic conditions (Figure 3k).

## 3. Discussion

**Figure 4:**
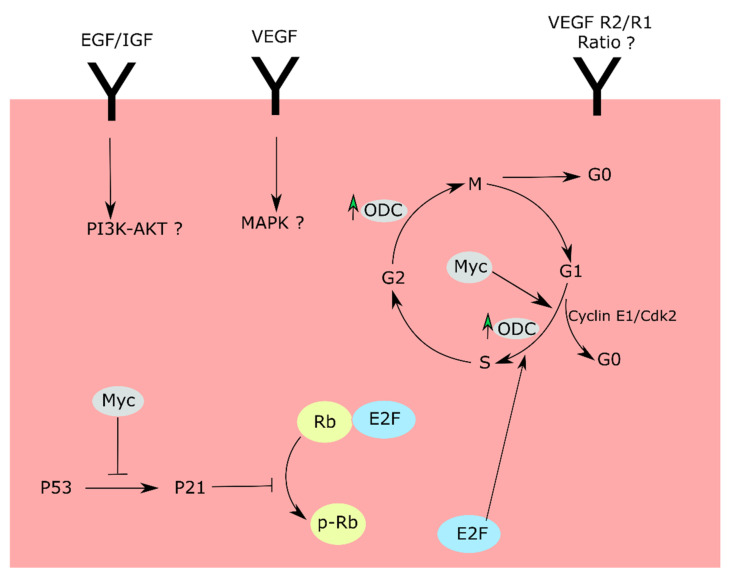
Cell-cycle checkpoints and proposed routes affected by hyperoxia. Hyperoxia downregulates Myc and upregulates P53 proteins, thereby increasing p21 protein levels. P21 further downregulates pRb levels, leading to cell-cycle arrest in the G1 phase. The cell-cycle arrest can be due to anomalies in MAPK and P13K/Akt pathways downstream of VEGF, EFG/IGF receptors. Alternatively, the cell-cycle changes can be a result of an aberrant VEGFR2/R1 ratio.

Our results definitively demonstrate that hyperoxia downregulates endothelial cell proliferation, without inducing cell death, by decreasing expression of key cell-cycle determinants (Figure 4). Although cell proliferation is controlled by multiple mechanisms under physiological conditions, p53 and Myc are reported to be the most important regulators of cell proliferation. Myc controls expression of positive regulators of the cell cycle and also induces growth by downregulating the expression of cell-cycle inhibitors such as p21CIP1/WAF1 (for review, see Bretones, Delgado, and Leon (2015)) [22]. The most widely accepted and recognized mechanism of p21 repression by Myc is through Miz-1. Miz-1, when in contact with Myc, represses p21. Miz-1/Myc interaction also makes p21 insensitive to p53 signaling [41,42]. The significance of our observation that an oxygen-rich environment induces downregulation of Myc is that it demonstrates that the central paradigm of the inverse relationship of HIF and Myc expression may not hold true in hyperoxia. Downregulation of Myc in hyperoxia, when HIF1 levels are known to be decreased, is unexpected—as Myc, in most cases, works antagonistically to HIF [23,24,25]. This warrants further studies on how and why hyperoxia downregulates Myc. Both Myc and p53 control these mechanisms in response to cellular stress such as DNA damage or nutrient deprivation [19,43]. Biomass synthesis pathways such as serine/one-carbon and glutaminolysis involve Myc protein. These pathways were found to be altered by hyperoxia in our previous studies, which further confirms that Myc in addition to HIF is another target of hyperoxic insult [44,45,46].

A second important finding from our experiments is that standard, HIF-induced mitogens are unable to override oxygen-induced growth suppression, at least in retinal endothelial cells. VEGF and other mitogens are known to activate endothelial cell proliferation. In our experiments, hyperoxia was able to block cell proliferation of endothelial cells, despite the presence of mitogens such as VEGF, IGF, and EGF—which implies that hyperoxia inhibits cell proliferation by acting downstream of these targets. Both MAPK and PI3K-Akt pathways control cell-cycle progression and are downstream of VEGF and EGF/IGF. Our findings suggest the relevance of these downstream pathways to OIR. Another independent possibility is that the cells, in response to hyperoxia, have an aberrant VEFR2/R1 ratio, rendering them less sensitive to mitogens. However, how this controls Myc is still an important future direction to aid further understanding of how high oxygen or hyperoxia alone contributes to such a tightly regulated signaling process.

In conclusion, our investigation demonstrates that hyperoxia downregulates retinal endothelial cell proliferation by downregulating Myc protein levels and upregulating p53 protein levels. The schema in Figure 4 provides a summary of our findings and a potential blueprint for examining how hyperoxia induces retinal endothelial cell growth suppression.

## 4. Materials and Methods

### 4.1. Cell Proliferation Assay

Primary human retinal endothelial cells were purchased from Cell Systems (Kirkland, WA, USA) and used within 4–5 passages. Cells were maintained in endothelial cell medium from Cell Biologics (catalogue number H1168; Chicago, IL, USA). Cells were plated in black 96-well plates overnight and then incubated in a normoxic (21% oxygen) or hyperoxic (75% oxygen) incubator for 4–6 days. Cell proliferation was measured by using a CyQuant^TM^ NF cell proliferation assay kit from Invitrogen (Waltham, MA, USA), following the protocol provided with the kit. A SMOX inhibitor, MDL 72527, was spiked into the medium at a final concentration of 100 µM.

### 4.2. Edu and Hoechst Staining

Cells were plated into chamber slides with 1 mL of endothelial cell media and incubated in a 37 °C, 5% CO_2_ incubator. After 24 h of incubation, slides were maintained in normoxic or hyperoxic (75% oxygen) incubators. Slides were maintained in these conditions for another 24 h, after which the medium was exchanged with the 900 µL of fresh medium. Slides were treated with 100 µL of 100 mg/mL Edu. Slides were incubated back in the normoxic or hyperoxic incubator for an additional 5 h. After incubation, the medium was discarded and cells were treated with 4% PFA prepared in PBS, and slides were incubated at room temperature for 15 min. Slides were then washed twice with 3% BSA prepared in PBS. Following this step, slides were treated with 0.5% TritonX-100 prepared in PBS. Prepared Click-iT^TM^ reagents as per the protocol provided by the manufacturer (kit catalogue number C10640; Invitrogen, Waltham, MA, USA). TritonX-100 was removed and each slide washed with 3% BSA prepared in PBS. A measure of 250 µL of Click-iT^TM^ mixture was added per chamber of the slide and incubated in the dark at room temperature for 30 min. After 30 min, the reaction cocktail was removed and cells were washed once with 3% BSA prepared in PBS. An additional washing step with PBS was performed before staining with Hoechst staining. Hoechst staining was performed following protocol provided by the Edu Click-iT^TM^ kit. Slides were sealed using a Vectashield antifade mounding medium (Vector laboratories, Burlingame, CA, USA) and coved with a cover slip. Images were taken on Zeiss AxioImager Z1 fluorescent microscope with a 10× objective. Images were merged on Fiji Image J [34]. Quantitative measurement was performed using ImageJ software [35].

### 4.3. Protein Extraction from Cultured Cells

Cells were plated in 100 mm × 20 mm dishes (Corning Inc., Corning, NY, USA), coated with the attachment factor (Cell Systems, catalogue number 4Z0-201; Kirkland, WA, USA) and maintained in the media described above. Once the cells reached 70–80% confluency, plates were either incubated in either normoxic (21% oxygen) or hyperoxic (75% oxygen) incubators for the next 24 h. Both the incubators were set at 37 °C temperature and 5% CO_2_. After 24 h of exposure to different levels of oxygen, proteins were extracted from these cells. To extract the proteins, cells were briefly washed with normal saline, followed by addition of 300 µL of RIPA buffer containing Complete^TM^ protease inhibitor and phosphatase inhibitor (both from Roche, Basel, Switzerland). Cells were scraped with cell scrapers and transferred to 1.5 mL tubes. Cells were briefly sonicated and then spun down in a centrifuge at 15,000× *g* for 15 min at 4 °C. The supernatant was transferred to fresh tubes and stored at −80 °C until further use.

### 4.4. SDS-PAGE and Western Blotting

Protein concentration in the cell lysates was measured using BCA protein assay reagent (Pierce^TM^). Protein sample 15–20 µg was mixed with tris-glycine SDS loading dye and 20 mM DTT. Samples were heated at 94 °C for 3 min following centrifugation at 15,000× g at room temperature for 3 min. Supernatant 30 µL was loaded into each well of 4–20% or 12% tris-glycine Novex^TM^ WedgeWell^TM^ precast gel (Invitrogen, Waltham, MA, USA). Equal quantities of protein samples were loaded in all the wells of each individual gel. Proteins were separated at constant voltage of 150 V. Proteins were transferred from gel to 0.45-micron PVDF membrane (Millipore, Burlington, MA, USA) at 70 V for 2 h using wet-transfer in tris-glycine buffer. Following transfer, membranes were dried for 1 h then quickly rinsed with methanol, followed by rinsing with water. Membranes were then washed with TBS and blocked with intercept TBS-blocking buffer (LI-COR, Lincoln, NE, USA) for 1 h. Following blocking, membranes were treated with primary antibodies diluted in intercept TBS blocking buffer containing 0.2% Tween 20 overnight at 4 °C. Membranes were washed with TBST three times (5 min per wash) then treated with secondary antibodies diluted in intercept TBS blocking buffer containing 0.2% Tween 20 and 0.01% SDS (*w*/*v*) solution, for 1 h, at room temperature, in the dark. Following incubation with secondary antibody, blots were washed three times with TBST and rinsed with TBS. Images were acquired on Odyssey^®^ CLx imaging system (LI-COR, Lincoln, NE, USA). Images were analyzed using Image Studio Lite version 5.2 (LI-COR, Lincoln, NE, USA)

It has earlier been noted previously that the Myc antibodies bind to a nonspecific band that co-elutes with endogenous Myc [47]. We also observed the nonspecific band that eluted very closely with Myc. To circumvent this problem, we included an additional step of stripping and reprobing the blot for Myc protein. This step was necessary to remove a second nonspecific band seen in our Myc blots. A p21 western blot was stripped with 10 mL of Restore^TM^ PLUS western blot stripping buffer (Thermo Fisher Scientific, Waltham, MA, USA) for 20 min at room temperature followed by reprobing with Myc and β-actin antibody for 1 h at room temperature. After treatment with primary antibody, the above-described procedure was used for secondary antibody treatment and imaging.

The following primary antibodies were used:

(1) c-Myc (D84C12) Rabbit mAb catalog # 5605
(2) Phospho-Rb (Ser 807/811) (D20B12) XP^®^ Rabbit mAb catalog # 8516
(3) Phospho-Rb (Ser 795) Rabbit antibody catalog # 9301
(4) p21 Waf1/Cip1 (12D1) Rabbit mAb catalog # 2947
(5) p53 Rabbit antibody catalog # 9282
(6) βactin (8H10D10) Mouse mAb catalog # 3700

All the antibodies were purchased from Cell Signaling (Danvers, MA, USA) and were diluted as recommended by the vendor. The following secondary antibodies were used:

(1) IRDye^®^ 800CW Donkey (polyclonal) anti-Rabbit IgG (H+L), catalog number 925-32213 from LI-COR (Lincoln, NE, USA).
(2) IRDye^®^ 680RD Donkey (polyclonal) anti-mouse IgG (H+L), catalog number 925-68072 from LI-COR (Lincoln, NE, USA).

Both the antibodies were used at 1:2000 dilution.

### 4.5. RNA Extraction and Quantitative RT-PCR

Cells were cultured in 6-well plates and maintained in endothelial cell medium in normoxic incubator. At around 70–80% confluence, cells were transferred to a normoxic or hyperoxic incubator for 24 h, as described above. Following this, RNA was extracted using TRI reagent (Sigma-Aldrich, St. Louis, MO, USA) using the protocol provided with the reagent. The RNA was converted into cDNA using Verso cDNA synthesis kit (Thermo Fisher Scientific, Waltham, MA, USA). Two µL of this cDNA was mixed with 10 µL of 2× qPCR mix Radiant^TM^ SYBR Green Lo-ROX (Alkali Scientific, Pompano Beach, FL, USA), 1 µL of 10 µM forward (Fwd) primer, 1 µL of 10 µM reverse (Rev) primer and 6 µL of nuclease free water. PCR settings were 50 °C for 2 min, 95 °C for 10 min, then 40 cycles at 95 °C for 15 sec and 60 °C for 1 min. Following PCR completion, a melting curve was recorded using these settings: 95 °C for 15 sec, 60 °C for 1 min, and 95 °C for 15 sec.

Sequences of the primers used for RT-PCR:
SMOX Fwd 5′-TCAAAGACAGCGCCCAT-3′SMOX Rev 5′-CCGTGGGTGGTGGAATAGTA-3′PAOX Fwd 5′-ACTAGGGGGTCCTACAGCTA-3′PAOX Rev 5‘-CGTGGAGTAAAACGTGCGAT-3′

### 4.6. Metabolite Extraction

Retinal endothelial cells were plated in 100 mm dishes coated with attachment factor (Cell Systems, Kirkland, WA, USA) at densities of 0.9 or 0.4 × 10^6^ cells per plate, and maintained in endothelial cell medium (Cell Biologics, Chicago, IL, USA) in a 5% CO_2_ incubator set at 37 °C for 3 days. After 3 days of incubation, the medium was changed to high-glucose DMEM medium (Cleveland Clinic Media lab, Cleveland, OH, USA) without FBS, and cells were again incubated in normoxic incubator, to synchronize the cells. After 6 h, the medium was changed back to endothelial cell medium (Cell Biologics, Chicago, IL, USA) and plates were either incubated in normoxic or hyperoxic (75% oxygen), 5% C0_2_ incubator set at 37 °C for 24 h. Following 24 h of incubation, metabolites were extracted. To extract metabolites, the medium was aspirated, and plates were washed with 10 mL of room-temperature normal saline. To the washed cells, 300 µL of 0.1% formic acid (prepared in water) containing 1 µg of ^13^C_5_ ribitol per ml of solution was added. Next, 600 µL of −20 °C cold methanol was added to each plate. Cells were scraped with a cell scraper while the plates were kept on ice, and cell lysates were transferred to tubes containing 450 µL of −20 °C cold chloroform. Tubes were agitated on a thermomixer at 4 °C, 1400 rpm, for 30 min. Tubes were then centrifuged for 5 min at 15,000× g at 4 °C. Six-hundred microliters of supernatant were transferred to fresh tubes and dried under vacuum in a −4 °C cold vacuum evaporator (Labconco, Kansas City, MO, USA). Samples were derivatized with the two-step protocol as described earlier (Singh et al., 2020, [45] and measured using the GCMS method described earlier (Singh et al., 2020, [46]).

### 4.7. Statistical Analysis

Statistical analyses of qPCR, western blot, cell culture, and metabolomics were performed by comparing means using the two-tailed Student’s t-test in Graphpad Prism software (GraphPad Software, San Diego, CA, USA). Error bars in all figures represent standard deviation (SD). Bar graphs were plotted in Graphpad Prism software.

## Supplementary Materials

The following are available online at https://www.mdpi.com/article/10.3390/life11070614/s1, Figure S1: Full view of Western blot analysis of Myc; Figure S2: Full view of Western blot analysis of p21; Figure S3: Full view of Western blot analysis of p35; Figure S4: Full view of Western blot analysis of pRB795; Figure S5: Full view of Western blot analysis of pRB807811.
