# Supplementary figures and images for: Hyperoxia Inhibits Proliferation of Retinal Endothelial Cells in a Myc-Dependent Manner
Source: Life (Basel). 2021 Jun 25;11(7):614. doi: 10.3390/life11070614 (PMC8304924; doi:10.3390/life11070614)

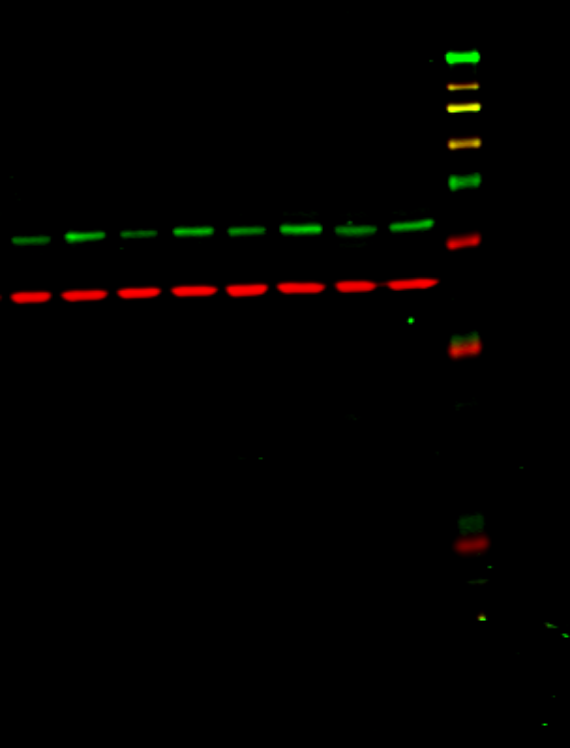

Supplement: Supplementary file 1 [file life-11-00614-s001.zip › Figure S1 - Myc.png]

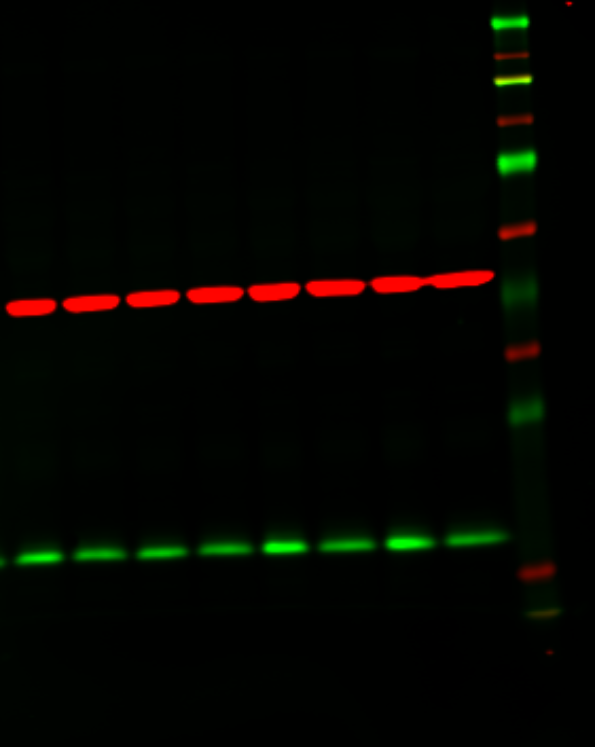

Supplement: Supplementary file 1 [file life-11-00614-s001.zip › Figure S2 - p21.png]

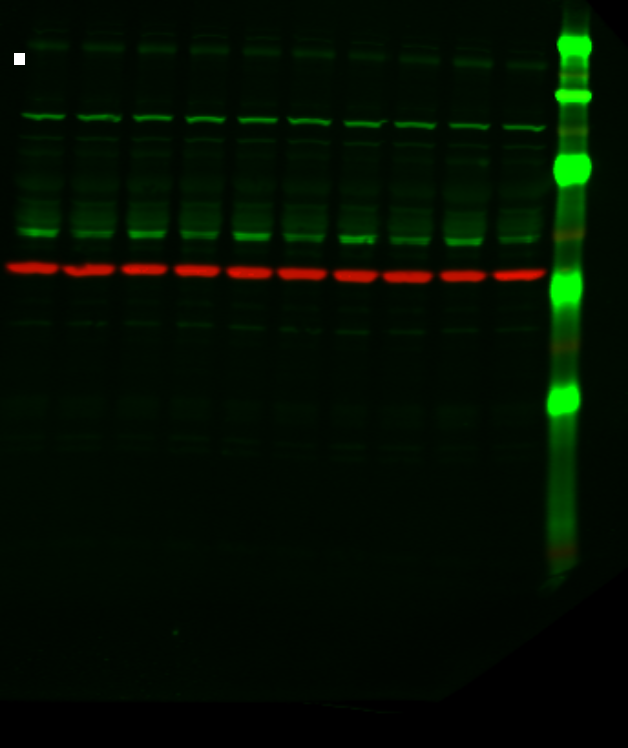

Supplement: Supplementary file 1 [file life-11-00614-s001.zip › Figure S3 - p53.png]

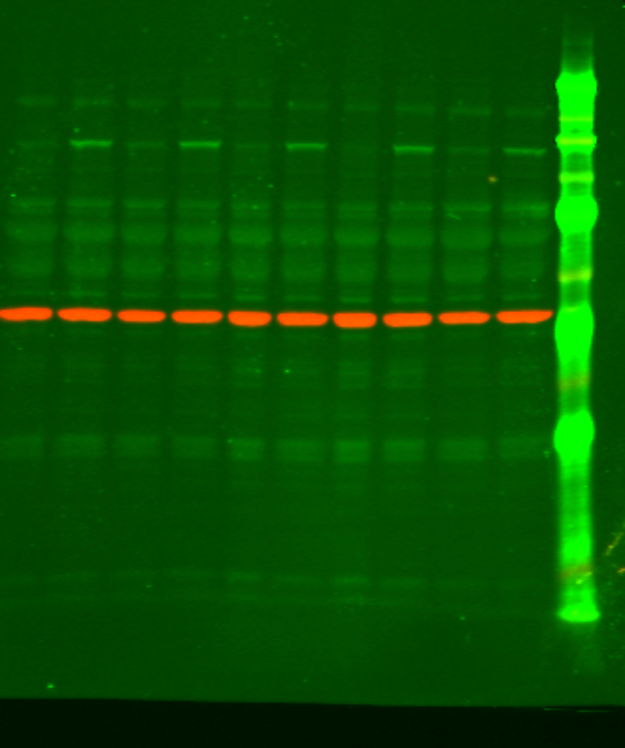

Supplement: Supplementary file 1 [file life-11-00614-s001.zip › Figure S4 -pRB795.png]

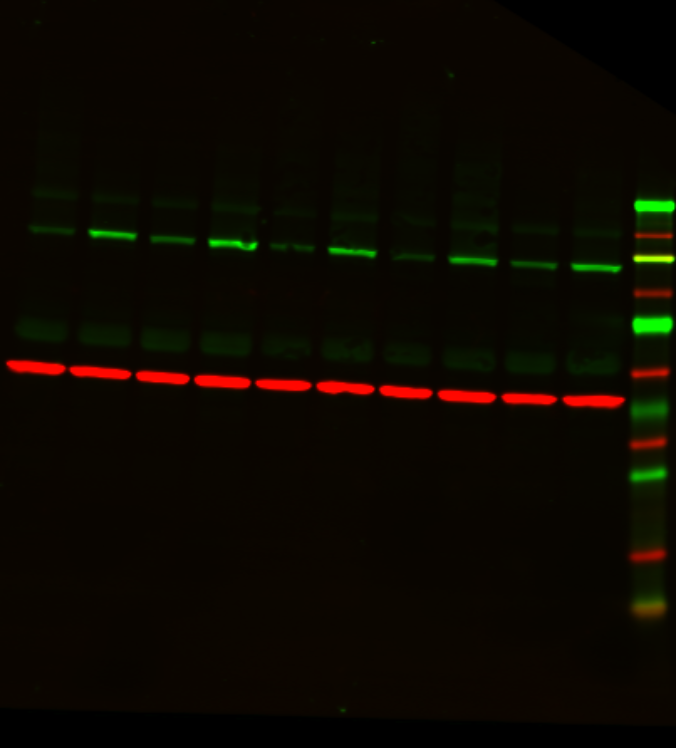

Supplement: Supplementary file 1 [file life-11-00614-s001.zip › Figure S5 -pRB807811.png]
